# Bandgap engineering of Tb-doped CoNiFe_2_O_4_-rGO composites for photocatalytic dye degradation

**DOI:** 10.1016/j.isci.2026.116539

**Published:** 2026-06-22

**Authors:** Tehmina Kousar, Yasmeen G. Abou El-Reash, Basem E. Keshta, Muhammad Aadil, Qurshia Choudhry, Mohamed N. Goda, Sahar Abdalla, Hany Koheil, Muhammad Farooq Warsi, Farzana Mahmood, Anish khan, Khalid A. Alzahrani, Eida S. Al-Farraj, Ahmad Hosseini-Bandegharaei, Ahmed M. Abodif

**Affiliations:** 1 Institute of Chemical Sciences, Bahauddin Zakariya University, Multan 60800, Pakistan; 2 Department of Chemistry, College of Science, Imam Mohammad Ibn Saud Islamic University (IMSIU), P.O. Box 90950, Riyadh 11623, Saudi Arabia; 3 Chemistry Department, Faculty of Science, Tanta University, Tanta 31527, Egypt; 4 Institute of Chemistry, Baghdad-ul-Jadeed Campus, The Islamia University of Bahawalpur, Bahawalpur 63100, Pakistan; 5 Physics and Engineering Mathematics Department, Faculty of Engineering, Kafrelsheikh University, Kafrelsheikh 33516, Egypt; 6 Center of Excellence for Advanced Materials Research, King Abdulaziz University, Jeddah, Saudi Arabia; 7 Chemistry Department, Faculty of Science, King Abdulaziz University, Jeddah, Saudi Arabia; 8 Faculty of Chemistry, Semnan University, Semnan, Iran; 9 Scientific Research Center, Al-Ayen Iraqi University (AUIQ), Nasiriyah, Dhi-Qar 64001, Iraq; 10 Civil Engineering Department, Higher Institute of Engineering and Technology, New Minia 61111, Egypt

**Keywords:** Industrial processing of material, Materials science

## Abstract

This study developed a magnetically recoverable, solar-driven photocatalyst for methylene blue (MB) dye mineralization. Three materials were synthesized: cobalt-nickel ferrite (CNFO), terbium-doped CNFO (Tb-CNFO), and a reduced graphene oxide composite (Tb-CNFO@rGO), using wet-chemical and ultrasonication methods. Structural, optical, compositional, and photocatalytic properties were evaluated. X-ray diffraction confirmed a cubic spinel phase with crystallite sizes of 16, 22, and 19 nm for CNFO, Tb-CNFO, and Tb-CNFO@rGO, respectively. Tauc plot analysis indicated bandgap reductions from 2.98 eV (CNFO) to 2.28 eV (Tb-CNFO) and 1.53 eV (Tb-CNFO@rGO). UV-Vis spectroscopy showed enhanced visible-light absorption for Tb-CNFO@rGO. EPR analysis identified hydroxyl (·OH) and superoxide (O_2_^−^) radicals as dominant species. Tb-CNFO@rGO exhibited superior photocatalytic activity, with a rate constant of 0.030 min^−1^, significantly higher than CNFO and Tb-CNFO, and retained 85.6% activity after five cycles via facile magnetic separation. These results demonstrate Tb-CNFO@rGO as an efficient, reusable photocatalyst for solar-driven dye wastewater treatment.

## Highlights

• Tb doping narrows the CoNiFe_2_O_4_ bandgap from 2.98 to 2.28 eV
• RGO hybridization further reduces the bandgap to 1.53 eV
• Magnetic catalysts retain 85% activity after five reuse cycles
• Composite degrades methylene blue at 0.030 per minute under sunlight

## Graphical abstract

**Figure:**
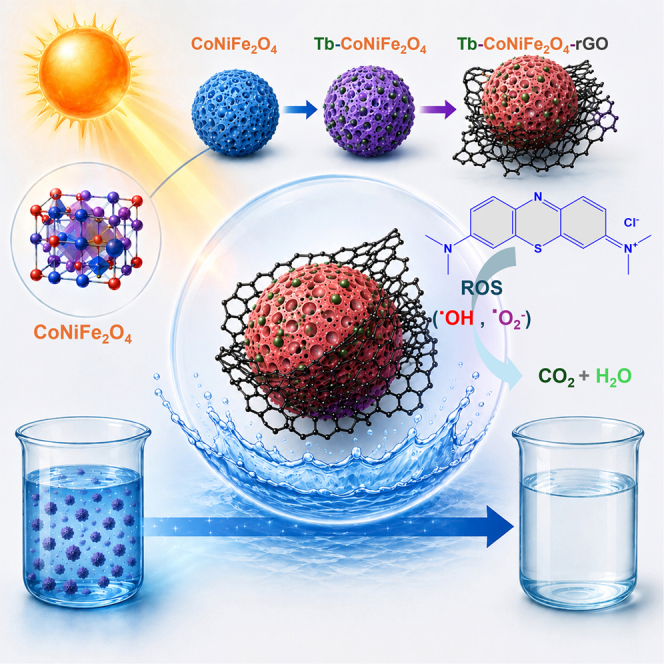

## Introduction

The rapid expansion of modern industrial activities, particularly in the textile, leather, paper, and food sectors, has resulted in the widespread discharge of synthetic dye-laden effluents into natural water bodies.^1^^,^^2^^,^^3^^,^^4^ Global dye production currently exceeds several hundred thousand tons annually, a substantial fraction of which is lost as wastewater during dyeing processes. These effluents are characterized by complex aromatic molecular structures, high chemical oxygen demand, and pronounced resistance to conventional biological and physicochemical degradation.^5^^,^^6^^,^^7^ Among synthetic dyes, cationic species such as MB have attracted particular attention owing to their demonstrated mutagenicity, potential carcinogenicity, and adverse effects on aquatic ecosystems and human health, including impairment of liver, kidney, and central nervous system function.^1^^,^^8^ Removal of persistent organic contaminants from contaminated water is thus a critical environmental and public health priority.^9^^,^^10^^,^^11^

Conventional wastewater treatment methodologies, including coagulation-flocculation, adsorption onto activated carbon, membrane separation, and aerobic biodegradation, suffer from notable limitations. These include incomplete pollutant mineralization, generation of secondary waste streams, high operational costs, and limited effectiveness toward persistent organic compounds that resist biological breakdown.^1^^,^^2^^,^^12^ These shortcomings have stimulated increasing research interest in advanced oxidation processes (AOPs), which exploit the generation of highly reactive oxygen species (ROS), primarily hydroxyl radicals (·OH) and superoxide radical anions (O_2_^−^) to achieve complete or near-complete mineralization of organic contaminants into CO_2_, H_2_O, and inorganic ions.^13^^,^^14^^,^^15^ Among AOPs, heterogeneous photocatalysis has emerged as a particularly attractive strategy owing to its capacity to harness solar energy, its operational simplicity, and its potential for scalable implementation in environmentally sensitive contexts.^16^

Spinel ferrites, with the general formula MFe_2_O_4_ (M = Co, Ni, Zn, Cu, Mn, etc.), constitute an important class of multifunctional semiconductor materials that have attracted increasing interest as magnetically recoverable photocatalysts.^17^^,^^18^^,^^19^^,^^20^^,^^21^ Their structural versatility stems from the ability to accommodate a broad range of metal cations at both tetrahedral (A-site) and octahedral (B-site) positions within the cubic close-packed oxygen sublattice, enabling systematic tuning of electronic, optical, and magnetic properties.^22^ In comparison with conventional wide-bandgap photocatalysts such as TiO_2_ (Eg ≈ 3.2 eV) and ZnO, spinel ferrites offer the distinct advantage of a narrower optical bandgap (∼1.9–2.5 eV), promoting absorption of visible and near-visible solar irradiation (SR). Moreover, their inherent ferrimagnetism enables facile recovery and reuse by an applied external magnetic field, circumventing the costly and energy-intensive solid-liquid separation procedures required by non-magnetic alternatives.^23^ Mixed spinel ferrites, such as cobalt-nickel ferrite (Co_0_._5_Ni_0_._5_Fe_2_O_4_, CNFO), combine the high magnetic anisotropy of CoFe_2_O_4_ with the improved crystallinity and electrical conductivity of NiFe_2_O_4_, yielding materials with enhanced photocatalytic profiles relative to their single-metal counterparts.^22^

Despite these advantages, bare spinel ferrite nanoparticles are limited in photocatalytic efficiency by two interrelated factors: rapid recombination of photogenerated electron-hole pairs and insufficient visible-light harvesting capacity. To address these limitations, the incorporation of graphene-family nanomaterials, particularly reduced graphene oxide (rGO) as a support matrix, has emerged as a highly effective strategy.^24^^,^^25^^,^^26^^,^^27^ The two-dimensional architecture of rGO, characterized by a high theoretical specific surface area (∼2630 m^2^ g^−1^), exceptional electrical conductivity, and delocalized π-electron conjugation, functions synergistically with ferrite nanoparticles in multiple ways.^28^ First, rGO serves as an efficient electron acceptor and transporter, rapidly extracting photogenerated electrons from the ferrite conduction band and suppressing electron-hole recombination. Second, the strong π–π interactions between rGO sheets and the aromatic chromophoric structures of dye molecules promote preferential adsorption of pollutants at the catalyst surface, thereby enhancing the effective concentration of target molecules in proximity to active photocatalytic sites. Third, the coupling of the ferrite and rGO electronic structures introduces recent interfacial states that extend optical absorption into the longer-wavelength visible region.^28^ The combination of these features significantly enhances the activity of ferrite/rGO nanocomposites compared to each component for solar-driven dye degradation.

A complementary strategy for enhancing the photocatalytic performance of spinel ferrites involves substitutional doping with rare-earth (RE) elements. The unique electronic configuration of lanthanide ions, characterized by partially occupied 4f orbitals shielded by outer 5s and 5p shells, confers several functional advantages when incorporated into ferrite lattices.^29^^,^^30^^,^^31^^,^^32^ The introduction of localized mid-gap 4f energy states creates shallow electron traps that reduce band-to-band recombination and prolong charge carrier lifetimes. Additionally, the large ionic radii of RE^3+^ ions relative to Fe^3+^ induce controlled lattice distortion at B-sites, modifying the local crystal field and exerting a downward shift on the optical band gap through hybridization of the 4f states with the host Fe 3d and O 2p orbitals. Elements such as lanthanum (La), cerium (Ce), europium (Eu), praseodymium (Pr), dysprosium (Dy), and terbium (Tb) have all been investigated as dopants in spinel ferrite photocatalysts, consistently yielding improved visible-light response and dye degradation rates relative to undoped counterparts.^29^^,^^30^ Terbium (Tb^3+^), in particular, possesses a distinctive 4f^8^ electronic configuration with a sizable magnetic moment and well-separated 4f energy levels, making it a strategically promising dopant for bandgap engineering in mixed spinel systems.^33^

The simultaneous deployment of RE doping and rGO hybridization in a single composite architecture offers a synergistic route to overcoming the intrinsic limitations of spinel ferrite photocatalysts. Rare-earth doping narrows the bandgap and improves charge separation at the bulk level, while rGO integration enhances surface reactivity, electron transport, and light harvesting at the interfacial level. Several recent studies have demonstrated the superiority of such bifunctional composites over their singly modified analogues in the photocatalytic degradation of organic dyes and emerging contaminants.^33^ Nevertheless, research specifically exploring Tb^3+^ as the rare-earth dopant in combination with rGO within a mixed Co-Ni spinel ferrite matrix and evaluating the resulting composite under natural solar irradiation remains limited, representing a clear gap in the current literature that the present study seeks to address.

In this work, we report the design, synthesis, and comprehensive characterization of Tb^3+^-doped cobalt-nickel mixed spinel ferrite (Tb-CNFO) and its rGO composite (Tb-CNFO@rGO) as magnetically recoverable, solar-active photocatalysts for the degradation of MB dye. The materials were synthesized via a microemulsion-assisted wet-chemical route combined with ultrasonication-assisted assembly, and characterized using X-ray diffraction (XRD), SEM, EDX, FTIR, and UV-Vis spectroscopy. The photocatalytic performance was evaluated under natural SR, with kinetic analysis, radical-trapping experiments using electron paramagnetic resonance (EPR), and recyclability assessment carried out systematically. The influence of Tb^3+^ incorporation and rGO hybridization on bandgap narrowing, charge-carrier dynamics, and dye mineralization efficiency is discussed in relation to the material’s structural and optical properties. This study examines Tb^3+^ as a bandgap engineering dopant in a mixed NiCoFe_2_O_4_/rGO composite system, achieving a bandgap of 1.53 eV and superior solar-driven photocatalytic performance via a simple microemulsion route, distinguishing this work from existing graphene-ferrite photocatalysts.

## Results

### XRD analysis

**Figure 1:**
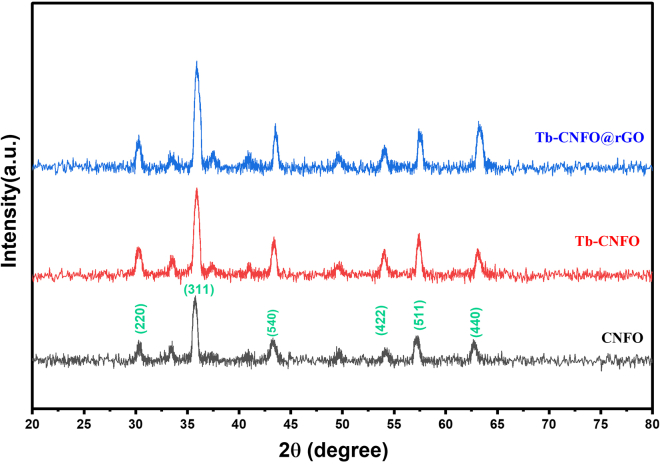
XRD analyses of CNFO, Tb-doped CNFO, and Tb-CNFO@rGO

Figure 1 is shown the XRD patterns of all synthesized samples, CNFO, Tb-CNFO, and CNFO@rGO, display clear peaks that are characteristic of the cubic spinel structure, indicating high crystallinity and the successful formation of the spinel phase. The main diffraction peaks can be attributed to the (220), (311), (400), (422), (511), and (440) planes, as per JCPDS card no. 33–0661.^34^ Doping with Tb^3+^ ions in Tb-CNFO causes a slight shift of diffraction peaks and a marginal reduction in lattice parameter from 8.34 Å (CNFO) to 8.32 Å (Tb-CNFO). While Tb^3+^ (0.923 Å) has a larger ionic radius than Fe^3+^ (0.645 Å) which would classically predict lattice expansion the observed marginal contraction at low doping levels (x = 0.06) is attributed to Tb^3+^ induced cation redistribution between A and B sites and local lattice strain relaxation, whereby the host matrix accommodates the ionic misfit through bond angle distortion rather than uniform unit cell expansion, a behavior previously reported for low-concentration RE^3+^ doped spinel ferrites. Decreased crystallite size and possible strain caused by graphene integration might be factors in the peak widening seen in the CNFO@rGO composite. The absence of extra peaks indicates phase purity and the effective inclusion of dopants and graphene without the creation of secondary phases. The lack of distinct rGO peaks (the broad band around ∼24–26° 2θ or the residual peak near ∼10–11° 2θ) is linked to the low rGO loading (approximately 1 wt %) in the Tb-CNFO@rGO composite. At these low concentrations, rGO is extensively exfoliated and evenly distributed within the ferrite matrix, leading to inadequate crystalline ordering for detection by XRD.

The average diameters of the produced sample materials were determined using the renowned Scherrer Equations 1^35^^,^^36^^,^^37^:

(Equation 1) $$D=\frac{K\lambda }{\beta \;\mathrm{COS}\;\theta }$$

The average particle sizes obtained from the samples are presented in Table 1, and they closely correspond to the particle sizes calculated from the SEM pictures. This table delineates the structural and physical parameters of three samples: CNFO (Co_0_._5_Ni_0_._5_Fe_2_O_4_), Tb-CNFO (Terbium-doped), and CNFO@rGO. Lattice constant (a), cell volume, crystallite size, X-ray density, specific surface area, dislocation density, and lattice microstrain were examined for each sample. Tb doping and rGO integration cause a little drop in the lattice constant (a) and unit cell volume. This implies that Tb^3+^ ions (with a lower ionic radius than Fe^3+^) might replace Fe^3+^ in the crystal lattice, hence causing lattice deformation and contraction. Possibly because of grain expansion after calcination, Tb doping increased the crystallite size from 16 nm (CNFO) to 22 nm. The incorporation of rGO, however, reduced the crystallite size to 19 nm, likely due to suppressed grain growth or surface stress caused by graphene layers. Progressive X-ray density throughout the sample points to a denser crystalline structure upon doping and composite creation. The highest density, 41.19 g/cm^3^, was found in CNFO@rGO, suggesting better crystallinity and compaction. From 9.11 (CNFO) to 13.00 (CNFO@rGO), SSA rose noticeably, which is good for photocatalytic uses as a larger surface area enables more active sites. Tb doping (0.00206 nm^−2^) shows a drop in dislocation density; rGO integration (0.00277 nm^−2^) shows a modest rise due to interfacial strain. Especially in the Tb-doped sample, lower dislocation density indicates fewer structural flaws and improved crystalline quality. Furthermore, lattice microstrain evaluated using the Williamson-Hall method reveals that Tb-CNFO exhibits lower microstrain than pristine CNFO, confirming improved crystallinity, whereas Tb-CNFO@rGO shows higher microstrain due to stress and defect formation at the ferrite-graphene interface. In contrast, rGO increases surface area, and Tb doping increases crystallinity; a structural study shows that both are beneficial for photocatalytic performance.

**Table 1:** The obtained average particle sizes

Sample	Crystal Lattice parameter a (A^o^)	Cell Volume (A^o^)	Crystallite Size (nm)	X-ray Density (gcm^−3^)	Specific Surface Area (per unit)	Dislocation density	Lattice Microstrain, ε
CNFO	8.34	581.2	16	24.31	9.11	0.00390625	4.89 × 10^−3^
Tb-CNFO	8.32	576.8	22	37.76	10.30	0.002066116	2.14 × 10^−3^
CNFO@rGO	8.31	575.5	19	41.19	13.00	0.002770083	5.58 × 10^−3^

The XRD derived structural parameters directly rationalize the photocatalytic activity trend (CNFO < Tb-CNFO < Tb-CNFO@rGO): the progressive increase in specific surface area (9.11 → 13.00 m^2^/g) enlarges the density of accessible active sites, the reduction in lattice parameter (8.34 → 8.31 Å) confirms Tb^3+^-induced lattice distortion responsible for bandgap narrowing and mid-gap 4f state formation, the decreased dislocation density in Tb-CNFO (2.07 × 10^−3^ nm^−2^) reflects improved crystallinity that reduces bulk recombination centers, and the elevated microstrain in Tb-CNFO@rGO (5.58 × 10^−3^) reflects productive interfacial strain at the ferrite–rGO junction that facilitates rapid electron extraction into the graphene network collectively accounting for the superior rate constant of 0.030 min^−1^ achieved by Tb-CNFO@rGO.^38^ The patterns revealed support effective CoNiFe_2_O_4_ alteration by means of doping and nanocomposite techniques. The computed lattice parameter roughly aligns with the values reported in the literature.^39^^,^^40^^,^^41^

### Morphology analysis

**Figure 2:**
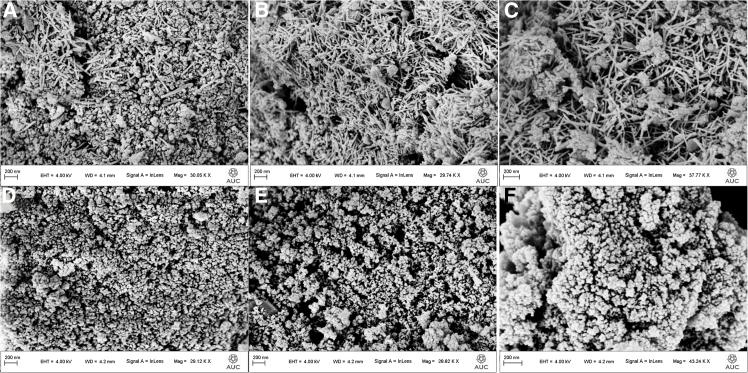
Morphological characterization of the synthesized catalysts (A–C) CNFO. (D–F) Tb-CNFO.

The dimensional characteristics, surface roughness, and shape of ferrite nanoparticles (CNFO and Tb-CNFO) made by wet chemical synthesis were studied by SEM, as illustrated in Figure 2. CNFO’s SEM pictures show a rather consistent, tightly packed granular structure. Though showing a clear porosity structure, the particles are clustered. Ranging in size from nanometers, the grains seem closely linked, implying a great surface area, which is good for catalytic uses. A little rough surface roughness can improve dye adsorption during photocatalytic activity. A considerable change in surface morphology is seen with Tb doping. Compared to pure CNFO, the Tb-CNFO picture shows a finer agglomeration structure. Larger Tb^3+^ ions, which disrupt the lattice and influence nucleation during synthesis, seem to make the grains look somewhat bigger and more distinct. The improved surface roughness and higher porosity resulting from Tb doping are both beneficial for active site exposure and overall photocatalytic performance. By lowering agglomeration and increasing porosity, Tb doping leads to heterogeneous nucleation, fine agglomeration, and reduced growth of particles, changes the surface shape overall, hence boosting photocatalytic performance by increasing dye molecule access and light absorption.^42^ The average grain size of CNFO and Tb-CNFO was calculated using the line intercept method (LIM) and ImageJ software,^43^ Equation 2.

(Equation 2) $$Particle\;size=\frac{1.5L}{MN}$$

Where L is the total number of lengths used by the test line in the SEM micrographs, *N* the total number of intercepts, and *M* image magnification. SEM images reveal that pure CNFO consists of agglomerated nanoparticles with an average size of 85 nm. In contrast, Tb-CNFO exhibits smaller, more uniformly dispersed particles with an average size of 62 nm, indicating that Tb doping inhibits grain growth and reduces agglomeration, consistent with previous reports on rare-earth-doped spinel ferrites.^44^

### FTIR analysis

**Figure 3:**
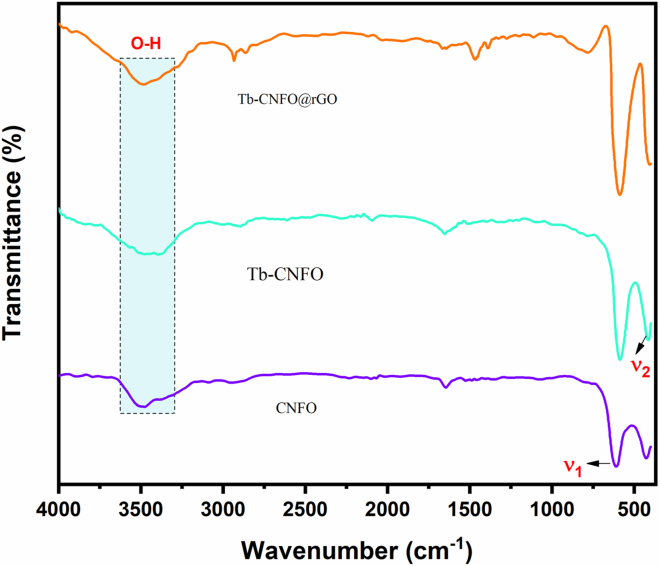
FTIR of undoped and Tb-doped CNFO samples

The FTIR spectra of nanoparticles are acquired within the range of 4000 to 400 cm^−1^, while the major vibration modes of ferrite materials are commonly found below 800 cm^-1.^^45^^,^^46^^,^^47^^,^^48^ The FTIR spectra of CNFO, Tb-CNFO, and CNFO@rGO samples were recorded to establish the presence of functional groups and validate the creation of spinel ferrite and its composite. All samples exhibited a broad absorption band centered at approximately 3400 cm^−1^, attributed to the O–H stretching vibrations of surface-adsorbed water molecules. A signal at 1600 cm^−1^ was ascribed to H–O–H bending vibrations,^49^ further suggesting surface hydration. The unique absorption bands at ∼580 cm^−1^ and ∼450 cm^−1^ are diagnostic of metal-oxygen vibrations in the spinel ferrite structure, corresponding to tetrahedral ⱱ_1_ (A-site) and octahedral ⱱ_2_ (B-site) M–O stretching, respectively. These peaks demonstrate the effective synthesis of the CoNiFe_2_O_4_ ferrite phase.^50^ Figure 3 unequivocally shows typical vibrational bands linked to the spinel ferrite structure and composite production. Especially, the (ⱱ_1_) band shows a little blue shift (higher frequency) when the material moves from CNFO to Tb-doped CNFO. By contrast, the (ⱱ_2_) band exhibits a red shift (lower frequency), and in the Tb-CNFO sample, this peak becomes much weaker or almost nonexistent, perhaps owing to a shift beyond the observable spectral window.^51^ This spectrum behavior implies that replacement of terbium (Tb^3+^) at the octahedral Fe^3+^ sites alters the local lattice environment. Their inclusion raises the site radius at the B-site as Tb^3+^ ions have a bigger ionic radius (0.923 Å) than Fe^3+^ ions (0.645 Å), therefore reducing the vibrational frequency in line with the known inverse correlation between site radius and vibrational mode energy. Tb^3+^ doping-induced Fe^3+^–O^2-^ bond distortion accounts for the observed change in ⱱ_2_ and disturbance in ⱱ_1_,^52^ which results in partial structural reordering or potential orthorhombic TbFeO_3_ phase creation at greater Tb concentrations.^53^ The Tb-CNFO@rGO composite stretching vibration peaks were detected at roughly 1128 cm^−1^, 1580 cm^−1^, and 1630 cm^−1^, which are linked to C-O stretching, C=C skeletal vibrations, and C=O stretching modes, respectively.^54^^,^^55^ This verifies the effective inclusion of graphene layers, hence improving the surface area and electrical characteristics of the hybrid nanostructure. The coexistence of ferrite and graphene vibrational characteristics confirms the development of the Tb-CNFO@rGO nanocomposite, which may have consequences for enhanced photocatalytic activity and electron transport efficiency.

### EDX analysis

**Figure 4:**
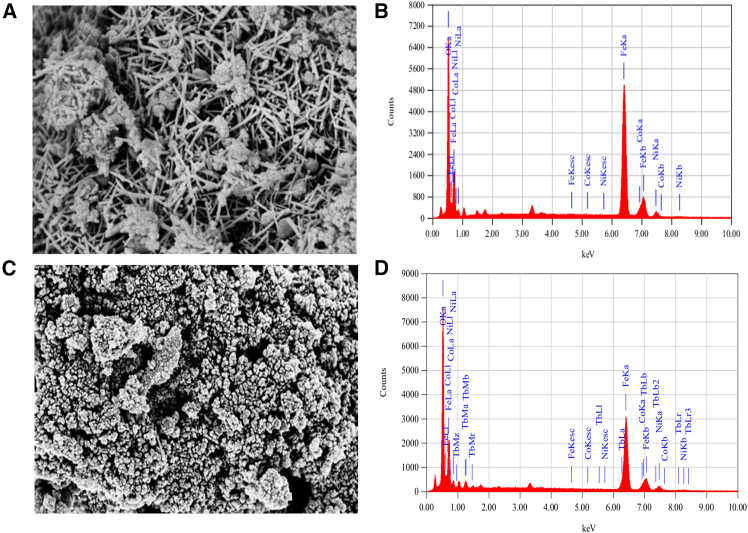
EDX spectrographs characterization of the synthesized catalysts (A and B) CNFO. (C and D) Tb-CNFO.

The image shows the SEM microstructures and related EDX spectra of the undoped cobalt-nickel ferrite (CNFO) and the terbium-doped version (Tb-CNFO). The analysis is described in a table, and the EDX spectrum is displayed in Figure 4. The precise stoichiometric ratio of Ni-C o-Fe- Tb-O ferrite is 0.5: 0.5: 2-x: x: 4, which is the atomic ratio of Ni, Co, Fe, Tb, and O. The presence of contaminants and other substances is unfounded. The spectra provided show that the synthesized samples are pure because there is no impurity peak.^56^

Indicative of polycrystalline ferrite particles, the SEM micrograph (a) shows an agglomerated microstructure with clear grain boundaries and a somewhat dense morphology. Fundamental to the spinel Co_0_._5_Ni_0_._5_Fe_2_O_4_ structure, the EDX spectrum (b) verifies the existence of important elements Co, Ni, and Fe. There are further peaks for oxygen (O) and small background signals confirming the oxide character of the material. The b1 EDX spectrum for the Tb-doped material shows extra clear peaks related to Tb, hence verifying the effective inclusion of Tb^1^ ions into the host lattice. Peaks for TbLa, TbLb1, TbLb2, and TbLγ confirm many electronic transitions of terbium in the material. The increased peak intensities for Fe and the emergence of Tb peaks imply B-site replacement of Fe^3+^ ions, which is in line with ionic radius compatibility and previous structural analyses. The related SEM Figure 4C of the Tb-CNFO sample reveals a more spread out morphology with linked grain structures. Lattice distortion or surface energy changes brought on by terbium inclusion might cause this, since they can affect sintering behavior and grain development during production.

Overall, Table 2 shows the energy dispersive X-ray spectroscopy (EDX) elemental analysis of cobalt-nickel ferrite nanoparticles (Co_0_._5_Ni_0_._5_Fe_2_O_4_), in undoped (x = 0) and terbium-doped (x = 0.06) compositions. The information comprises the atomic and mass percentages of the identified elements. With a mass% of 77.76 and atom% of 57.14, iron (Fe) predominates the composition of the undoped sample, corresponding well with its stoichiometric abundance in the spinel ferrite structure. Oxygen (O) records as 14.65% by mass, therefore reflecting the oxide character of the substance. Indicating appropriate inclusion into the A-sites of the spinel lattice, cobalt (Co) and nickel (Ni) show up in modest but constant amounts, 3.28% and 4.31% by mass, respectively.

**Table 2:** EDX data of Tb-CNFO Ferrites nanoparticles

Sample	Element	Mass%	Atom%
Tb-CNFO Ferrites nanoparticles for x = 0	O K	14.65	37.57
	Fe K (Ref.)	77.76	57.14
	Co K∗	3.28	2.28
	Ni K	4.31	3.01
	Total	100	100
Tb-CNFO Ferrites nanoparticles for x = 0.06	O K (Ref.)	22.59	52.14
	Fe K	62.23	41.16
	Co K∗	2.95	1.85
	Ni K	5.08	3.19
	Tb L∗	7.16	1.66
	Total	100	100

Terbium doping causes significant variations in the elemental distribution. By mass, the oxygen concentration rises to 22.59% and by atoms to 52.14%, indicating more oxygen coordination, probably caused by lattice strain or Tb–O bond formation. Consistent with the doping process, iron concentration drops markedly to 62.23% (mass%), suggesting partial replacement of Fe^3+^ by Tb^3+^ ions. With 7.16% mass and 1.66% atomic percentage, terbium is obviously found, verifying its effective incorporation. There are also slight changes in Co and Ni concentrations that stay near predicted levels, suggesting that the replacement mostly impacts the B-site (occupied by Fe). Overall, the addition of Tb confirms replacement by lowering the Fe concentration. Enhanced oxidation states or changed lattice dynamics might lead to more O content. Presence of Tb (7.16 mass%) verifies effective doping and supports the structural changes deduced from XRD and FTIR data.

### Energy band gap studies

**Figure 5:**
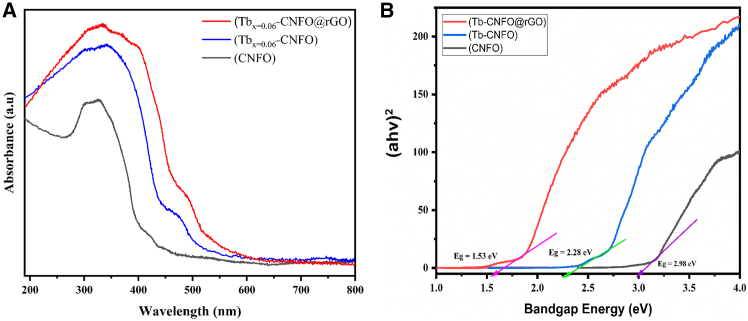
The UV-Vis absorption spectra of the prepared catalysts (A) UV-Vis absorption of CNFO, Tb-CNFO, and Tb-CNFO@rGO. (B) Tauc Plot of CNFO, Tb-CNFO, and Tb-CNFO@rGO.

UV-Vis’s absorption spectroscopy and Tauc plot analysis were used to evaluate the optical characteristics of CNFO, Tb-CNFO, and Tb-CNFO@rGO nanocomposites, as indicated in Figure 5. Doping and composite creation show a distinct increase in visible light absorption, as shown in the UV-Vis absorption spectra. Pure CNFO exhibits relatively weak absorption in the visible region, consistent with its modest photocatalytic activity under solar irradiation. Doping with terbium causes the Tb-CNFO sample to absorb more noticeably, implying that the addition of rare-earth ions changes the electronic structure, perhaps by adding 4f levels interacting with the Fe 3d and O 2p orbitals. The Tb-CNFO@rGO composite shows the most notable improvement since strong π–π conjugation results from the synergistic interaction between the graphene sheets and Tb-doped spinel ferrite matrix, therefore enabling better light-harvesting capacity across the UV and visible areas. The Equation 3, which describes the Tauc plot approach, was also used to determine the optical band gap energy of the synthesized product.

(Equation 3) $${\;\left(\alpha h\nu \right)}^{2}=A\left(h\nu -E_{g}\right)$$

The variables in the equation are defined as follows: α represents the molar absorptivity, h represents Planck’s constant, v represents the frequency of light, A represents the absorbance, and E_g_ is the bandgap energy. The bandgap energy may be determined by extrapolating the linear portion of the curve displayed between “hν” (on the *x* axis) with (α hν)^2^ (on the *y* axis). The related Tauc graphs show a gradual drop in optical bandgap energies, hence supporting this trend even more. Characteristic of a low visible light reaction, CNFO has a large bandgap of 2.98 eV. While adding graphene reduces the bandgap to 1.53 eV, doping with Tb reduces it to 2.28 eV. This significant red shift in bandgap shows how well Tb doping and graphene integration work to shape the electrical band structure, therefore allowing effective use of the solar spectrum. These findings provide strong evidence that Tb-CNFO@rGO is rather appropriate for visible-light-driven photocatalytic uses, such as dye degradation, and has improved optical characteristics.

The UV-Vis absorption spectra in Figure 5A serve as indirect optical evidence of charge-separation efficiency, complementing the photodegradation data. The progressively enhanced absorption intensity and broadened visible-light response from CNFO → Tb-CNFO → Tb-CNFO@rGO indicate that a greater photon flux is harvested per unit time under solar irradiation, generating a higher density of electron-hole pairs available for ROS production. Importantly, the pronounced absorption shoulder extending beyond 500 nm in Tb-CNFO@rGO is absent in bare CNFO, reflecting the synergistic contribution of Tb^3+^ 4f mid-gap states and rGO π-electron conjugation to sub-bandgap excitation. The Tauc plots in Figure 5B quantitatively confirm this trend, with bandgap values of 2.98, 2.28, and 1.53 eV for CNFO, Tb-CNFO, and Tb-CNFO@rGO respectively; this systematic red-shift directly accounts for the hierarchical improvement in rate constants (0.018 → 0.023 → 0.030 min^−1^), since a narrower bandgap permits excitation across a broader fraction of the solar spectrum, ultimately sustaining the ·OH and O_2_^−^ radical generation confirmed by EPR in photocatalytic mechanism section.

## Discussion

### Photodegradation of MB

**Figure 6:**
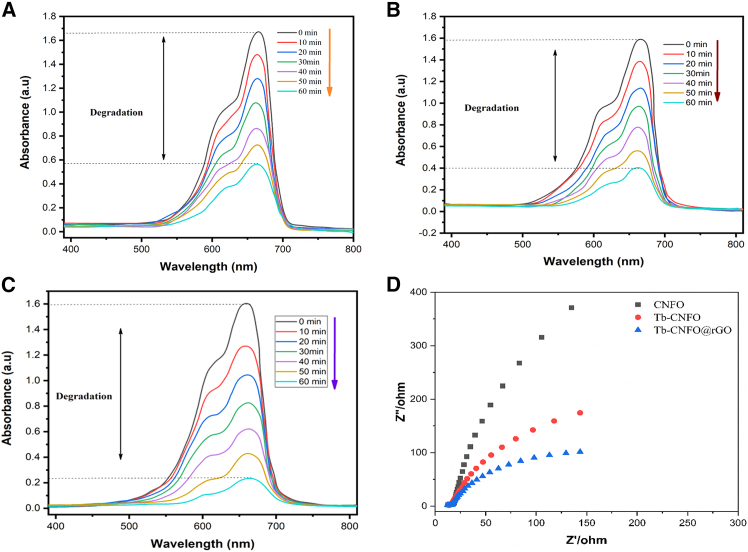
UV-Vis absorption of MB dye removal and electrochemical properties over several catalysts (A) CNFO. (B) Tb-CNFO. (C) Tb-CNFO@rGO. (D) EIS test results.

This study aims to examine the effectiveness of a nanocomposite consisting of Tb^3+^ modified Ni-Co ferrite and reduced rGO in the degradation of MB. In this experiment, a solution containing 20 mg/L of MB dye was combined with 50 mg of catalyst. The total volume of the solution used was 50 mL. The strength of the distinctive absorption peak of MB decreased over time, as shown in Figure 6. Figure 6 presents the UV-Vis absorption spectra demonstrating the degradation of MB dye over time using three different photocatalysts: CNFO, Tb-CNFO, and Tb-CNFO@rGO. Every graph indicates the degree of photocatalytic degradation under visible light irradiation by a gradual drop in the distinctive absorbance peak of MB dye (∼664 nm). A progressive drop in the MB absorbance intensity over time for the pure CNFO sample, Figure 6A, indicates modest photocatalytic activity. The Tb-doped CNFO Figure 6B reveals a more noticeable drop in absorbance, hence stressing the improvement in catalytic efficacy resulting from the addition of terbium ions. The insertion of 4f states from Tb^3+^ helps to better charge carrier separation and raises the production of ROS, hence improving this. The Tb-CNFO@rGO composite Figure 6C shows the most notable deterioration performance as the absorbance peak quickly drops over a shorter time period. The combined effects of Tb doping and graphene integration produce this better activity. Graphene not only enhances dye adsorption but also enables fast electron transport, hence reducing recombination of photogenerated charge carriers. This results in the generation of hydroxyl radicals, the main species causing dye mineralization. The graph generally shows clearly the hierarchical improvement in MB dye degradation performance: CNFO < Tb-CNFO < Tb-CNFO@rGO. These findings confirm the use of rare-earth doping and graphene reinforcement in designing high-performance photocatalysts for environmental remediation uses. In addition, the kinetic analysis showed that the decomposition rate of the MB dye was much higher (0.03048 min^−1^) in the nanocomposite containing rGO compared to the nanoparticles without any coating (0.02253 min^−1^).

The catalytic efficacy was linked to the efficiency of electron transfer across catalysts, and target pollutants. Electrochemical impedance spectroscopy (EIS) was utilized to evaluate the electron conductivity of the catalysts. The electrochemical characteristics of the synthesized catalyst composites, comprising CNFO, Tb-CNFO, and Tb-CNFO@rGO, were evaluated to determine their electron transfer efficiencies within the reaction system. Figure 6D illustrates that the diminished semicircle width in EIS signifies that Tb-CNFO, and Tb-CNFO@rGO display markedly lower electron-transfer resistance relative to CNFO.^1^ The curvature of composites Tb-CNFO@rGO, in comparison to Tb-CNFO and CNFO, indicates a minimal charge transfer resistance. The interaction between Tb and CNFO nanoparticles and rGO improves electron transport efficiency.

This study investigated the kinetics of the MB removal under the effects of different systems, solar photocatalytic (UV), CNFO, Tb-CNFO, and Tb-CNFO@rGO prepared catalysts. The investigation employed the pseudo-first-order kinetics model, which is described by Equation 4.

(Equation 4) ln (C_t_/C_o_) = −k_t_

**Figure 7:**
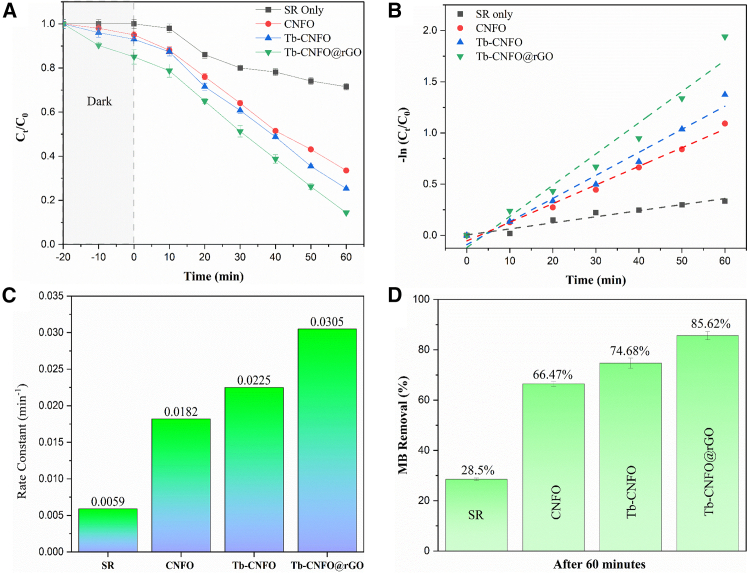
The degradation performance of the prepared catalysts (A and B) Kinetics profiles of MB removal over UV, CNFO, Tb-CNFO, and Tb-CNFO@rGO. (C and D) k_app_ values for MB-dye degradation over UV, CNFO, Tb-CNFO, and Tb-CNFO@GO, and MB removal percent (MB 20 mg/L, pH 7, and catalyst dosage 50 mg). (A and D) The value of error bars was clculated using standard error of the mean (SEM) method.

Where C_o_ and Ct denote the initial and ultimate concentrations, respectively, of the MB, measured in milligrams per liter, at the beginning and at time “t”. Figure 7B displays a graph depicting the correlation between the natural logarithm of the ratio of concentration at time “t” to the beginning concentration, -ln(C_t_/C_o_), and time “t”. The statistical parameters, including slope, regression coefficient, and intercept, were derived by applying a linear model to the data points plotted on the graph. Figure 7A shows the kinetic profiles of MB dye degradation under visible light irradiation and employing three photocatalysts: CNFO, Tb-CNFO, and Tb-CNFO@rGO. Tracking the variation in normalized concentration (Ct/C_0_) of MB dye during irradiation time facilitates the evaluation of degradation kinetics, hence providing insights into the photocatalytic efficiency and reaction rates of the synthesized materials. The UV and CNFO systems exhibit the lowest rate of dye removal, indicative of their weakened photocatalytic activity. A significant increase in the degradation rate is seen with Tb-CNFO as shown in Figure 7D, which may be ascribed to the addition of Tb^3+^ ions. These rare earth ions create localized 4f electronic states that increase the spacing of photogenerated electron-hole pairs, hence generating reactive species like hydroxyl radicals. The Tb-CNFO@rGO composite demonstrates the most significant enhancement, as the reaction kinetics indicate a more pronounced decrease in MB concentration within a reduced time interval. The synergistic impact of terbium doping and rGO integration is mostly responsible for this improved performance. The rGO speeds charge transfer and reduces recombination by means of its outstanding electron mediation. Moreover, its large surface area encourages improved dye adsorption, thus enhancing the photocatalytic reaction interface. The kinetic profiles show clearly that the Tb-CNFO@rGO nanocomposite has better photocatalytic efficiency than its undoped and non-graphene equivalents. These results highlight the possibility of rare-earth and graphene co-modified spinel ferrites in sophisticated wastewater treatment uses. The fitting results for the UV, CNFO, Tb-CNFO, and Tb-CNFO@rGO nanomaterials are displayed in the inset of Figure 7B.

The gradient of the fitted data provides a measure of the pace at which the MB dye photo-mineralization reaction occurs on a certain photocatalyst. The photo-mineralization rate constant (k) values under UV, CNFO, Tb-CNFO, and Tb-CNFO@rGO driven MB degradation processes were 0.0059 min^−1^, 0.018 min^−1^, 0.023 min^−1^, and 0.030 min^−1^, respectively, as indicated in Figure 7C. The improved mineralization activity of the Tb-CNFO@rGO nano ferrite in MB dye is attributed to its larger surface area, which allows for better light absorption and greater transfer of charge carriers.

To contextualize the photocatalytic performance of the present system, Table 3 compares the MB degradation efficiency and rate constant of Tb-CNFO@rGO with recently reported analogous spinel ferrite-based photocatalysts under similar conditions.

**Table 3:** Comparison of photocatalytic MB degradation performance

Photocatalyst	Conc. (mg/L)	Catalyst dose (mg/L)	Light source	Degradation (%)	k (min^−1^)	Cycles	Reference
Tb-CNFO@rGO (this work)	15	1000	solar	85.6	0.030	5	this work
rGO/TiO_2_/NiFe_2_O_4_	10	500	UV-Vis	95.0	–	5	Jihad et al.^57^
g-C_3_N_4_/GO/CuFe_2_O_4_	10	200	solar	91.4	0.031	4	Tahir et al.^58^
α-Fe_2_O_3_/rGO	5.34	400	visible	94.0	–	–	Anuradha et al.^59^

As shown in Table 3, Tb-CNFO@rGO achieves a competitive rate constant of 0.030 min^−1^ under natural solar irradiation with a low catalyst dose, while offering the additional advantages of magnetic recoverability and phase-pure spinel synthesis via a cost-effective microemulsion route, distinguishing it from several higher-performing but synthetically complex or UV-dependent systems.

### Photocatalytic mechanism

**Figure 8:**
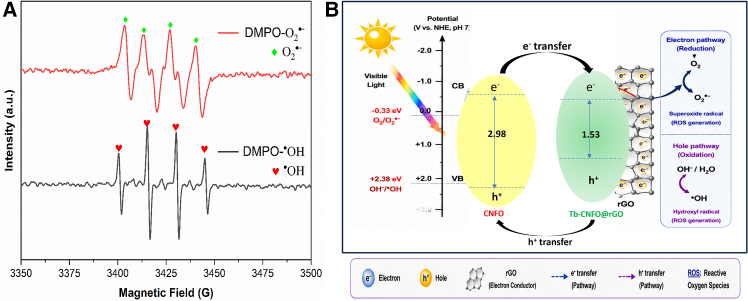
Trapping of reactive oxygen species and the activation mechanism (A) EPR test for Tb-CNFO@rGO. (B) The possible mechanism of photodegradation and electron transfer pathway via Tb-CNFO@rGO (MB 20 mg/L, pH 7, and catalyst dosage 50 mg).

The EPR method was employed to determine the essential radicals ·OH and O_2_·^−^ involved in the photocatalytic process.^60^ Following 20 min of visible light exposure, four separate peaks were found, each exhibiting peak intensities in a ratio of roughly 1:2:2:1. DMPO (5,5-dimethyl-1-pyrroline N-oxide) was used as the spin-trapping agent at a concentration of 50 mM in aqueous dispersion of Tb-CNFO@rGO under visible light irradiation. This observation validates the presence of ·OH in the system. The EPR spectrum Figure 8A exhibits four prominent and two minor peak intensities characteristic of DMPO O_2_·^−^, indicating the existence of O_2_·^−^ radicals in the degradation process.

The photocatalytic mechanism of MB degradation over Tb-CNFO@rGO is presented in Figure 8B and is directly governed by the optical bandgap characteristics established in section 3.5. Under SR, incident photons with energy equal to or exceeding the bandgap of Tb-CNFO@rGO (1.53 eV) are absorbed at a substantially lower threshold than bare CNFO (2.98 eV), confirming that Tb^3+^ doping and rGO hybridization together enable efficient excitation across a broad fraction of the solar spectrum. This excitation promotes electrons (e^−^) from the valence band (VB) to the conduction band (CB) of Tb-CNFO, generating electron-hole (e^−^/h^+^) pairs. The Tb^3+^ 4f mid-gap states, responsible for the bandgap reduction from 2.98 to 2.28 eV, act as shallow electron traps that temporarily localize photogenerated electrons, suppressing direct band-to-band recombination and prolonging carrier lifetime. The photogenerated electrons are subsequently transferred to the rGO network, which, owing to its exceptional electron mobility and π-conjugated structure, acts as an efficient electron sink, further inhibiting e^−^/h^+^ recombination as evidenced by the 1.7-fold improvement in rate constant over bare CNFO. The accumulated electrons on rGO reduce adsorbed molecular oxygen to superoxide radical anions O_2_·^-^, Equation 5, while the VB holes simultaneously oxidize surface hydroxyl ions and water molecules to hydroxyl radicals ·OH, Equation 6. Both ·OH and O_2_·^−^, confirmed as the dominant ROS by EPR spectroscopy Figure 8A, attack the chromophoric structure of MB, cleaving the aromatic rings and azo linkages (–N=N–) and mineralizing the dye into CO_2_, H_2_O, and inorganic ions Equation 7. The mechanistic hierarchy is thus: bandgap narrowing (UV-Vis, energy band gap studies section) → enhanced carrier generation → rGO-mediated charge separation → ROS production (EPR, Figure 8A) → MB mineralization (kinetics, Figure 7), with each step directly supported by the characterization data presented in this study.

(Equation 5) O_2_ + e^−^ → O_2_·^−^

Concurrently, the holes (h^+^) in the VB oxidize surface hydroxide ions (OH^−^) or water molecules, resulting in the formation of extremely reactive ·OH, Equation 6:

(Equation 6) h^+^ + OH^−^ → ·OH ^-^

Both O_2_·^−^ and ·OH radicals possess potent oxidative capability and attack the chromophoric structure of MB, leading to cleavage of the azo linkages (–N=N–) and progressive degradation of the organic molecule into smaller intermediates. Ultimately, these intermediates are converted into CO_2_ and H_2_O, Equation 7.

(Equation 7) MB + ·OH / O_2_·^−^ → CO_2_ + H_2_O + other mineralized products

The Tb-CNFO@rGO heterostructure improves photocatalytic efficiency via the synergistic effects of rare-earth (Tb^3+^) doping and rGO-facilitated charge separation, hence optimizing solar energy use and enhancing dye degradation efficacy. Tb-CNFO@rGO exhibits superior performance overall, hence endorsing the design strategy aimed at enhancing photocatalytic activity through rare earth doping and graphene hybridization. Tb-CNFO@rGO is a promising magnetically retrievable photocatalyst for efficient dye mineralization in environmental remediation.

### Reuse of photocatalyst

**Figure 9:**
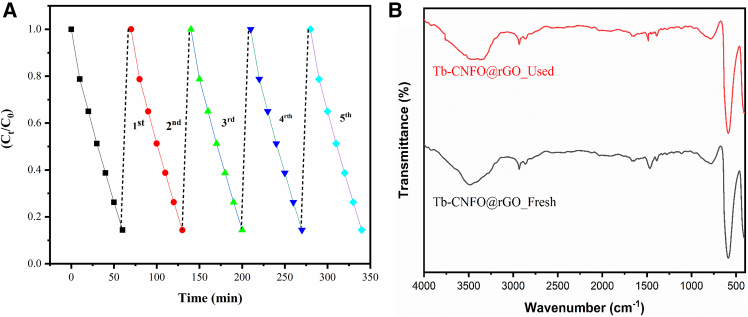
Reusability and structural stability (A) Stability recyclability of Tb-CNFO@rGO. (B) FTIR spectrum for fresh and used Tb-CNFO@rGO. (MB 20 mg/L, pH 7, and catalyst dosage 50 mg).

An excellent catalyst should possess two crucial characteristics: photochemical stability and reusability. After the photocatalytic process is finished, the ferrite materials may be fully and rapidly recovered due to their magnetic properties.^61^^,^^62^ The durability and recyclability of the Tb-CNFO@rGO nanomaterial were observed after five cycles of MB dye degradation under sunlight. After each photocatalytic operation, the magnetic photocatalytic material Tb-CNFO@rGO was retrieved and subsequently employed for the subsequent MB degradation cycle. Figure 9 illustrates the photo-degradation capability of the Tb-CNFO@rGO material. The Tb-CNFO@rGO nanomaterial exhibits remarkable chemical stability and complete magnetic recovery, allowing it to maintain 85.3% of its original MB degrading activity even after undergoing five cycles. The recyclability data demonstrate that our synthesized Tb-CNFO@rGO nanomaterial is durable, thereby displaying significant promise for use in the field of contaminated water remediation. The post-cycle FTIR measurement, Figure 9B of Tb-CNFO@rGO, following five successive degradation cycles, verifies exceptional chemical stability. The spectra of the fresh and utilized catalyst are essentially indistinguishable, exhibiting approximately no loss or shift in metal-oxygen bands or rGO-related characteristics, so indicating that the composite withstands photo corrosion and preserves structural integrity under repeated sun irradiation and dye degradation conditions.

Post-cycle FTIR was specifically selected as the stability diagnostic for three scientifically grounded reasons. First, FTIR directly probes the integrity of the chemical bonding environment of the catalyst the preservation of the characteristic M–O tetrahedral (ν_1_, ∼580 cm^−1^) and octahedral (ν_2_, ∼450 cm^−1^) stretching bands in the used catalyst confirms that the spinel ferrite crystal structure is retained and that no phase decomposition, surface oxidation, or leaching of metal ions has occurred during repeated photocatalytic cycles. Second, the persistence of rGO-related bands at ∼1128, ∼1580, and ∼1630 cm^−1^ in the post-use spectrum confirms that the graphene network remains structurally intact and has not been oxidized back to GO or fragmented under solar irradiation, a critical concern for carbon-based composites subjected to prolonged oxidative photocatalytic conditions. Third, FTIR provides direct evidence that no significant dye adsorption or surface poisoning has occurred on the catalyst surface after five cycles, as the absence of new peaks attributable to MB or its degradation intermediates in the post-use spectrum confirms that the active sites remain unblocked and accessible. Together, these three lines of FTIR evidence collectively validate the chemical, structural, and surface stability of Tb-CNFO@rGO, providing a more direct and chemically informative assessment of reusability than recyclability data alone.

From a practical applicability standpoint, Tb-CNFO@rGO demonstrates three features relevant to real-world deployment. First, the composite retained 85.3% photocatalytic activity after five consecutive cycles with facile magnetic recovery, confirming operational stability under repeated use without significant performance loss. Second, the synthesis employs a simple, low-cost microemulsion route using commercially available precursors, presenting no inherent barrier to scale-up. Third, while the present study employed MB as a model pollutant under controlled conditions, real wastewater matrices contain competing ions, variable pH, and mixed contaminants that may influence performance; future work should therefore evaluate Tb-CNFO@rGO in actual textile effluents and assess its activity toward emerging contaminants such as antibiotics and endocrine-disrupting compounds to fully establish its practical remediation potential.

In summary Tb-CNFO and Tb-CNFO@rGO were effectively synthesized using an economical micro-emulsion technique. XRD validated the emergence of a pure cubic spinel phase with a crystallite size of around 19 nm, while SEM demonstrated a homogeneous distribution of nanoparticles over rGO sheets. The introduction of Tb^3+^ reduced the bandgap from 2.98 eV (CNFO) to 2.28 eV (Tb-CNFO) and subsequently to 1.53 eV (Tb-CNFO@rGO), facilitating a robust response to visible light. The combined function of Tb^3+^ in creating mid-gap 4f states for bandgap reduction, along with rGO promoting swift electron transfer and inhibiting recombination, led to a substantial improvement in photocatalytic efficacy. Tb-CNFO@rGO attained a degradation rate constant of 0.03048 min^−1^ for MB, resulting in an 85.6% elimination within 60 min of solar irradiation, significantly surpassing CNFO (42%) and Tb-CNFO (74.7%). Pseudo-first-order kinetic analysis yielded rate constants of 0.018, 0.023, and 0.030 min^−1^ for CNFO, Tb-CNFO, and Tb-CNFO@rGO, respectively, representing a 1.7-fold enhancement for the composite over bare CNFO and approximately 5-fold over UV photolysis alone (0.006 min^−1^), with the composite retaining 85.3% activity after five cycles, as confirmed by post-use FTIR. The composite retained 85.3% of its original photocatalytic activity after five consecutive cycles, enabled by facile magnetic separation. Ultimately, Tb-CNFO@rGO facilitates efficient, magnetic, visible-light photocatalytic degradation of wastewater dyes in a scalable manner.

### Limitations of the study

(1) MB used as a single model dye rather than real textile complex effluent; (2) *In situ* characterization and machine learning are indispensable for revealing the procedures that result in the formation of active sites, offering insights for future precise synthesis of catalysts.; (3) recyclability tested only over 5 cycles; and (4) no LCA/TEA or scale-up demonstration.

## Resource availability

### Lead contact

Further information and requests for resources and reagents should be directed to and will be fulfilled by the lead contact, Basem E. Keshta (basem.keshta@science.tanta.edu.eg).

### Materials availability

This study did not generate new unique materials, cell lines, or biological resources. All synthesized samples are available from the lead contact upon reasonable request.

### Data and code availability

#### Data

Data reported in this paper will be shared by the lead contact upon request.

#### Code

This paper does not report original code.

#### Other items

Any additional information required to reanalyse the data reported in this paper are available from the lead contact upon request.

## Acknowledgments

This work was supported and funded by the Deanship of Scientific Research at Imam Mohammad Ibn Saud Islamic University (IMSIU) (grant number IMSIU-DDRSP2602).

## Author contributions

Conceptualization, data curation, investigation, methodology, writing – original draft, T.K.; validation, visualization, funding, Y.G.A.E.-R.; project administration, conceptualization, validation, visualization, supervision, writing – review and editing, B.E.K.; experimental work and data acquisition, Q.C. and M.A.; validation, visualization, M.N.G., E.S.A.-F., and S.A.; supervision, validation, M.F.W. and F.M.; visualization and investigation, H.K.; validation, visualization, A.K. and K.A.A.; conceptualization, supervision, data curation, investigation, methodology, writing – original draft, A.M.A. and A.H.-B. All authors discussed the results, contributed to the writing or critical revision of the manuscript, and approved the final version for publication.

## Declaration of interests

The authors declare no competing interests.

## Declaration of generative AI and AI-assisted technologies in the writing process

Statement regarding the utilization of generative AI and AI-assisted technologies in the manuscript preparation process The author(s) did not utilize generative AI or AI-assisted technologies as data sources for this work; they were employed solely for language enhancement and in the editing and presentation processes.

## STAR★Methods

### Key resources table

**Table:** 

REAGENT or RESOURCE	SOURCE	IDENTIFIER
**Chemicals, and materials**		

Cobalt(II) nitrate hexahydrate	Sigma Aldrich	CAS: 10026-22-9
Nickel (II) nitrate hexahydrate	Sigma Aldrich	CAS:13478-00-7
Terbium(III)nitrate hexahydrate	Sigma Aldrich	CAS: 13451-19-9
Sodium hydroxide	Sigma Aldrich	CAS: 1310-73-2
Iron(III) nitrate	Sigma Aldrich	CAS: 7782-61-8
Methylene blue	Sigma Aldrich	CAS: 122965-43-9
1-butanol	Sigma Aldrich	CAS: 71-36-3
Paraffin oil	Sigma Aldrich	CAS: 8012-95-1
Sodium nitrate	Sigma Aldrich	CAS:7631-99-4
Potassium permanganate	Sigma Aldrich	CAS: 7722-64-7
Sulfuric acid	Sigma Aldrich	CAS: 7664-93-9
Hydrogen peroxide	Sigma Aldrich	CAS: 7722-84-1
Ammonia	Sigma Aldrich	CAS: 7664-41-7
Hydrazine hydrate	Sigma Aldrich	CAS: 10217-52-4
Graphite powder	Sigma Aldrich	CAS: 7782-42-5

**Software and algorithms**		

Origin 2021	Origin 2021	https://www.originlab.com
MDI Jade 9	Materials Data	https://www.materialsdata.com/prodjd.html
ImageJ software	SEM Data	https://imagej.en.softonic.com/

### Materials

Sigma Aldrich supplied all chemicals of greater than 99.99% purity and there was no reagent or chemical purification.

### Experimental model and study participant details

There are no experimental models used in the study.

### Method details

#### Synthesis of Tb-CNFO

According to the literature, with slight modifications,^47^^,^^48^^,^^63^ A 0.1 mol/L aqueous solution of Ni(NO_3_)_2_, Co(NO_3_)_2_, Fe(NO_3_)_3_, and Tb(NO_3_)_3_ was prepared using analytical-grade salts (Sigma Aldrich, ≥99.99% purity) and mixed in a 500 mL vessel at stoichiometric ratios corresponding to the target composition Tb-CNFO. The doping level x = 0.06 was selected following preliminary screening of x = 0, 0.02, 0.04, 0.06, 0.08, and 0.10, at which x = 0.06 yielded the highest MB degradation efficiency; higher concentrations resulted in reduced activity attributed to secondary phase formation and excessive lattice distortion. The solution was heated under magnetic stirring, and at 65 °C, a microemulsion phase consisting of 1-butanol, paraffin oil, and Triton X-100 (1:1:1, v/v/v) was added. The pH was then adjusted to 11 by dropwise addition of NaOH solution under continuous stirring, and the reaction was allowed to proceed for 120 min to complete precipitation. The precipitate was separated, repeatedly washed with ethanol and deionized water to neutral pH, and dried at 85 °C, followed by grinding and calcination at 675 °C for 5.5 h to obtain the spinel ferrite phase. The obtained Tb-doped cobalt-nickel mixed spinel-ferrite, named Tb-CNFO, where CNFO was synthesized under identical conditions without a Tb precursor. Electrochemical measurements were carried out on a CHI660E electrochemical workstation (Shanghai Chenhua, China) using a standard three-electrode configuration. An Ag/AgCl electrode (saturated KCl) and a Pt electrode were used as the reference and counter electrodes, respectively. Fluorine-doped tin oxide (FTO) glass coated with 20 μL of catalyst slurry was used as the working electrode. A 0.2 mol/L Na2SO4 aqueous solution (pH = 7.1) was employed as the electrolyte, and all measurements were conducted at 25°C.

#### Synthesis of rGO

Reduced graphene oxide (rGO) was prepared from natural graphite using a modified Hummers’ oxidation followed by hydrazine reduction.^64^^,^^65^ Graphite (1.5 g) and NaNO_3_ (1.5 g) were dispersed in concentrated H_2_SO_4_ (75 mL, 98%), KMnO_4_ (9 g) was added below 10 °C, the mixture was stirred (30 min in ice, then 48 h at room temperature), diluted with water (138 mL), terminated with H_2_O_2_ (30 mL, 30 wt %), washed with 6 wt % H_2_SO_4_ and water to neutral pH, and dried to obtain GO. GO dispersion (10 mL, 2 mg mL^−1^) was diluted to 100 mL, ultrasonicated for 30 min, then reduced with hydrazine hydrate (80 wt %, 5 μL) and aqueous ammonia (25 wt %, 35 μL; final hydrazine ≈1.3 mM) at 90 °C for 1 h under stirring, yielding a black rGO dispersion that was washed and dried at 120 °C for 24 h.

#### Synthesis of Tb-CNFO@rGO composite

The Tb-CNFO@rGO nanocomposite was prepared via an ultrasonication-assisted assembly method. 0.9 g of Tb-CNFO nanoparticles and 10 mg rGO were dispersed in 20 mL of deionized water. The suspension was ultrasonicated using a probe sonicator operating at 20 kHz and 200 W output power for 60 min (pulse mode: 5s on/5 s off) while maintaining the temperature below 25 °C. After homogenization, the composite was washed with deionized water and dried at 120 °C for 12 h. The dried powder was gently ground and calcined at 675 °C with a heating rate of 5 °C min^−1^, held for 4 h, and naturally cooled to room temperature to obtain the final Tb-CNFO@rGO nanocomposite. The rGO loading was fixed at approximately 1 wt % relative to the ferrite mass (10 mg rGO per 0.9 g Tb-CNFO), a ratio selected to ensure adequate surface coverage without blocking active ferrite sites.

#### Photocatalytic activity of Tb-CNFO@rGO composite

The photocatalytic activity was evaluated under natural SR (Multan, Pakistan; average solar intensity ∼850–950 W/m^2^; experiments conducted between 11:00 a.m. and 1:00 p.m. on clear sunny days in summer). A 50 mL aqueous solution of MB (20 mg/L, pH 7, unadjusted) was mixed with 50 mg of catalyst (catalyst dose = 1.0 g/L) in a 100 mL open glass beaker. Before irradiation, the suspension was magnetically stirred in the dark for 20 min to establish adsorption–desorption equilibrium, ensuring that any initial concentration drop was solely attributable to adsorption rather than photocatalysis. The solution was then exposed to direct sunlight under continuous magnetic stirring at 400 rpm. Aliquots of 3 mL were withdrawn at 0, 20, 40, 60, 80, and 100 min intervals, immediately centrifuged at 4000 rpm for 5 min to remove the catalyst, and the supernatant was analyzed by UV–Vis spectroscopy at the characteristic MB absorption wavelength of 664 nm. A control experiment without a catalyst confirmed negligible MB photolysis under SR alone (<5% decolorization in 60 min). All experiments were triplicated to validate that degradation is photocatalytically calculated using the following Equation 8:

(Equation 8) $$\text{Degradation}\;\left(\%\right)=\frac{C_{0}-C_{t}}{C_{0}}\times 100$$

where *C*_0_ and *C*_*t*_ are the initial and instantaneous concentrations of MB, respectively.

#### Characterizations

X-ray diffraction (XRD) was performed using a Bruker D8 Advance diffractometer (Bruker AXS, Germany) equipped with a Cu Kα radiation source (λ = 1.5406 Å, photon energy = 8.04 keV) operating at 40 kV and 40 mA. Measurements were conducted in Bragg-Brentano geometry with a 1D Lynx Eye detector, over a 2θ range of 10–80° with 0.02° step size and 10° min-1 scan rate. The instrumental angular resolution was approximately 0.01°. Phase identification and lattice-parameter refinement were carried out using MDI JADE 9 software, with crystallite size estimated via the Scherrer equation. Data were analyzed with an uncertainty of ±0.02° in peak position. The surface morphology and microstructure of the samples were examined using Scanning electron microscope SEM and EDX characterized using Benchtop Scanning Electron Microscope JCM-6000PLUS. Fourier transform infrared (FT-IR) spectra were collected on a Nicolet 6700 spectrometer (Thermo Fisher Scientific, USA) using the KBr pellet method with a resolution of 4 cm-1.The surface morphology and microstructure of the samples were examined using a Hitachi SU-8010 field-emission scanning electron microscope (FE-SEM). UV-Vis were recorded using a Shimadzu UV-3600i Plus spectrophotometer in the wavelength range of 300–800 nm. BaSO_4_ was used as the reflectance reference. The reflectance data were converted using the Kubelka-Munk function F(R), and the optical band gap (Eg) was estimated from Tauc plots. Electrochemical impedance spectroscopy (EIS) tests to prove the direct electron transfer in the reaction system. The detailed method is to use a three-electrode system, the electrolyte is 1 M sodium sulfate solution, the auxiliary electrode is a platinum electrode, the reference electrode is a saturated calomel electrode(SCE), and the working electrode is the research sample CNFO, Tb-CNFO, and Tb-CNFO@rGO. The scanning range is 0∼1.0V vs. SCE), and the scanning rate is 50 mV/s. The EIS test was carried out at an open circuit potential, the test frequency range is 1 Hz- 1 × 10^5^ Hz, and the AC amplitude is 5mv. Electron Paramagnetic Resonance (EPR) Test In order to determine the active species in the system, the Bruker X-band A200 instrument of Bruker Company in Germany was used for electron paramagnetic. DMPO was used as the spin-trapping agent for detecting HO⋅, ⋅O_2_^−^, and TEMP was used for trapping ^1^O_2_. The EPR spectra were collected under the following conditions: center field 3430 G, microwave frequency 9.86 GHz, microwave power 10 mW, modulation amplitude 1.0 G, Scan width 100 G, receiver gain 50 db, Scan time 30.06 s.

### Quantification and statistical analysis

Crystallite sizes were calculated using the Scherrer equation (D = Kλ/βcosθ). Lattice microstrain was determined by the Williamson–Hall method. Photocatalytic degradation efficiency was calculated as: Degradation (%) = (C_0_ − Ct)/C_0_ × 100. Pseudo-first-order rate constants (k) were obtained by linear fitting of ln(Ct/C_0_) vs. time, with R^2^ values reported for each fit. All photocatalytic experiments were performed in triplicate; results are expressed as mean values. Optical bandgap energies were determined from Tauc plots by extrapolating the linear portion of (αhν)^2^ vs. hν.
